# Supplementary material for: Edaravone activates the GDNF/RET neurotrophic signaling pathway and protects mRNA-induced motor neurons from iPS cells
Source: Mol Neurodegener. 2022 Jan 10;17:8. doi: 10.1186/s13024-021-00510-y (PMC8751314; doi:10.1186/s13024-021-00510-y)
Supplement: Supplementary file 1 — Additional file 1: Supplemental Fig. 1. (A) TUJ1+ miMNs from the iPSC1 line (day 7 of differentiation) show no detectable expression of the pluripotent stem cell marker (OCT4) and the oligodendrocyte lineage marker (O4). (B) TUJ1+ miMNs from the iPSC1 line were immunostained for the cholinergic neuron marker ChAT. Cytoplasmic signal of the ChAT protein was not detected in these miMNs at day 10 of differentiation, compared to cytoplasmic ChAT signal in more mature miMNs at day 20 of differentiation (Fig. 3A). (C) The iPSC line iPSC1 was differentiated by 3 daily transfections of NSA mRNA alone without using OSA mRNA and morphogenes. TUJ1+ NSA-induced neurons (day 30 of differentiation) express the glutamatergic neuron marker VGlut1, validating the VGluT1 antibody also used in Fig. 3E and supporting the requirement of Olig2 for MN induction. Cell nuclei were counterstained with DAPI (Bar = 20 μm). (D) Control miMNs from iPSC1 and iPSC3 iPSCs (referred to as control miMN 1 and 2, respectively) and ALS miMNs from iPSC2 and iPSC4 iPSCs (referred to as ALS miMN 1 and 2, respectively) were plated to the MEA plate at day 4 of differentiation. Their spontaneous spiking was recorded at indicated days in vitro (Fig. 4G). The number of active electrodes at indicated days in vitro did not show difference between control and ALS miMNs (3 technical replicates for each miMN line, linear regression with clustered data, ALS vs control miMNs). Supplemental Fig. 2. Reproducibility of two replicates from the transcriptomic analysis of miMNs with +/− edaravone treatment. (A) Heatmap clustering of RNA-Seq results from miMNs with +/− edaravone treatment (10 μM, 24 h, n = 2 for each group). Gene expression was calculated by reads per kilobase of transcript, per million mapped reads (RPKM). (B) A transcriptomic comparison between miMNs and differentiating cells at various days during compound-induced MN differentiation from human ESCs (Reference 39, GSE140747 from the GEO database). PCA plot [file 13024_2021_510_MOESM1_ESM.docx]

**
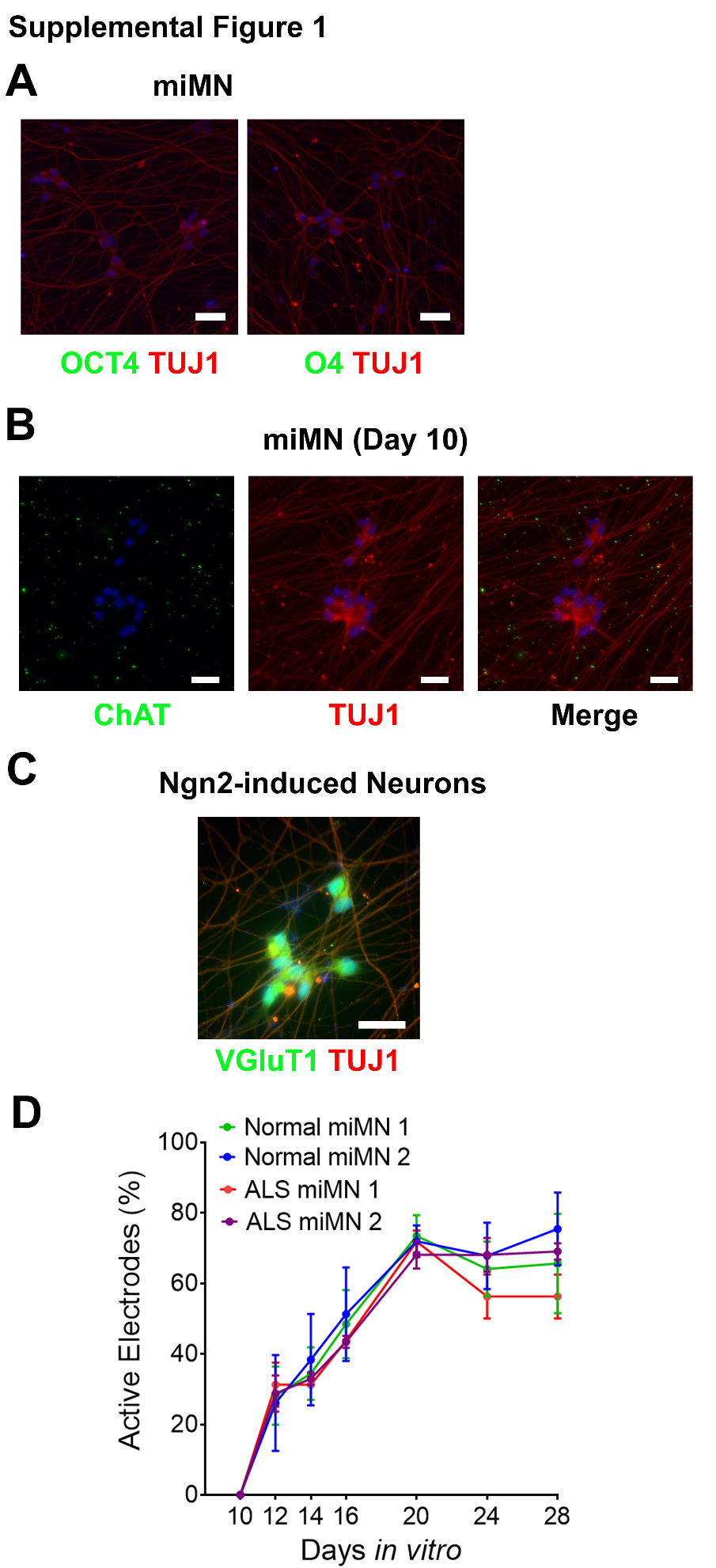
**

**Supplemental Figure 1.**

**(A)** TUJ1+ miMNs from the iPSC1 line (day 7 of differentiation) show no detectable expression of the pluripotent stem cell marker (OCT4) and the oligodendrocyte lineage marker (O4).

**(B)** TUJ1+ miMNs from the iPSC1 line were immunostained for the cholinergic neuron marker ChAT. Cytoplasmic signal of the ChAT protein was not detected in these miMNs at day 10 of differentiation, compared to cytoplasmic ChAT signal in more mature miMNs at day 20 of differentiation (Figure 3A).

**(C)** The iPSC line iPSC1 was differentiated by 3 daily transfections of NSA mRNA alone without using OSA mRNA and morphogenes. TUJ1+ NSA-induced neurons (day 30 of differentiation) express the glutamatergic neuron marker VGlut1, validating the VGluT1 antibody also used in Figure 3E and supporting the requirement of Olig2 for MN induction.

Cell nuclei were counterstained with DAPI (Bar = 20 μm).

**(D)** Normal miMNs from iPSC1 and iPSC3 iPSCs (referred to as normal miMN 1 and 2, respectively) and ALS miMNs from iPSC2 and iPSC4 iPSCs (referred to as ALS miMN 1 and 2, respectively), were plated to the MEA plate at day 4 of differentiation. Their spontaneous spiking was recorded at indicated days *in vitro* (Figure 4G). The number of active electrodes at indicated days *in vitro* did not show difference between normal and ALS miMNs (3 technical replicates for each miMN line, linear regression with clustered data, ALS vs normal miMNs).

**
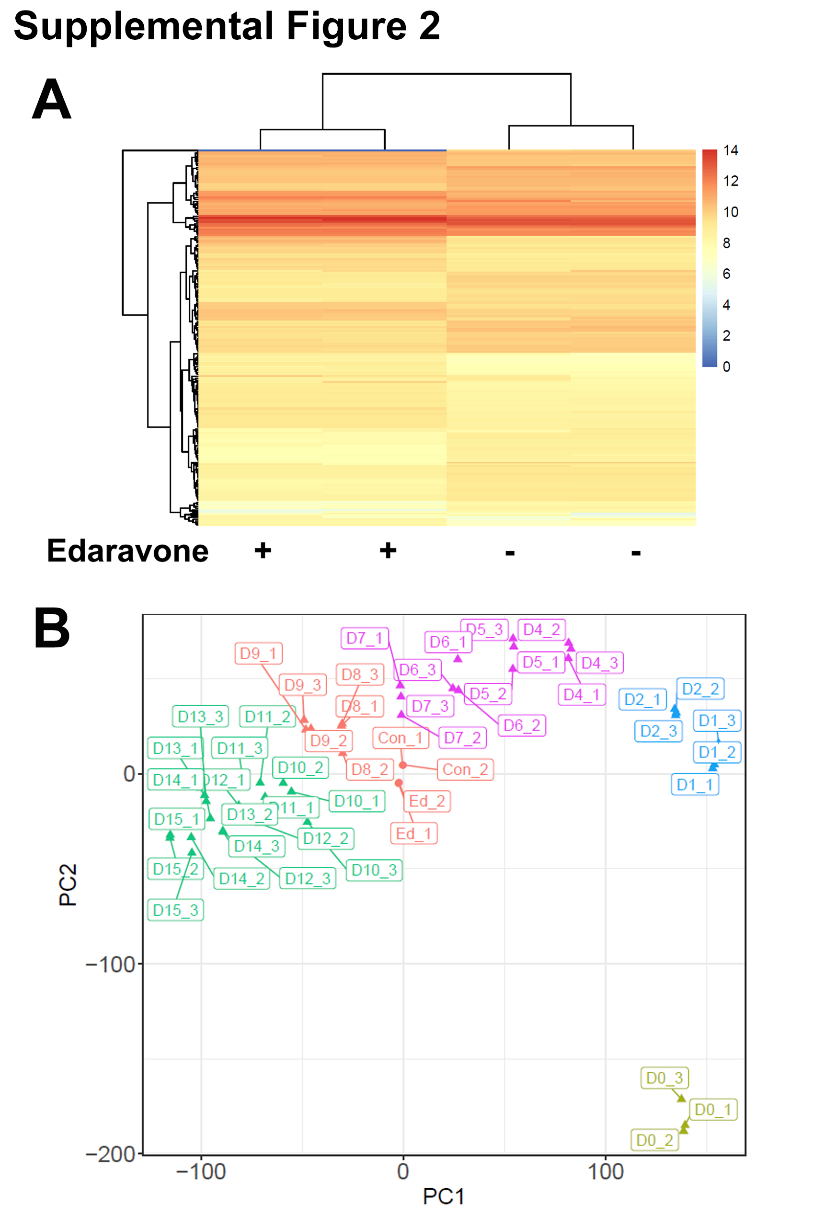
**

**Supplemental Figure 2. Reproducibility of two replicates from the transcriptomic analysis of miMNs with +/- edaravone treatment.**

**(A)** Heatmap clustering of RNA-Seq results from miMNs with +/- edaravone treatment (10 µM, 24h, n=2 for each group). Gene expression was calculated by reads per kilobase of transcript, per million mapped reads (RPKM).

**(B)** A transcriptomic comparison between miMNs and differentiating cells at various days during compound-induced MN differentiation from human ESCs (Reference 39, GSE140747 from the GEO database). PCA plotting shows control (Con_1, Con_2) and edaravone-treated (Ed_1 and Ed_2) miMN samples (two technical replicates) more closely cluster with compound-induced MNs at day 8 and 9 of differentiation (three technical replicates labelled as D8_1/D8_2/D8_3 and D9_1/D9_2/D9_3, respectively). All samples are labelled as day at differentiation (D)_replicate number. All samples are grouped and colored based on the clustering result using model-based clustering of RNA-seq data.

**Supplemental Tables:**

**Table S1.** Up-regulated genes in normal miMNs after edaravone treatment.

**Table S2.** Down-regulated genes in normal miMNs after edaravone treatment.

**Table S3.** Expression data of all genes detected by RNA-seq.

**Table S4.** IPA signaling pathways enriched in edaravone up-regulated genes.

**Table S5.** IPA signaling pathways enriched in edaravone down-regulated genes.

**Table S6.** qPCR primers and antibodies.
