# Supplementary material for: Edaravone activates the GDNF/RET neurotrophic signaling pathway and protects mRNA-induced motor neurons from iPS cells
Source: Mol Neurodegener. 2022 Jan 10;17:8. doi: 10.1186/s13024-021-00510-y (PMC8751314; doi:10.1186/s13024-021-00510-y)
Supplement: Supplementary file 7 — Additional file 7: Table S6. qPCR primers and antibodies. [file 13024_2021_510_MOESM7_ESM.pdf]

| Gene | Primer Sequence |
|---|---|
| **HB9** | GCCTAAGATGCCCGACTTCAAC |
| | CGCGACAGGTACTTGTTGAGCT |
| **Islet1** | GCAGAGTGACATAGATCAGCCTG |
| | GCCTCAATAGGACTGGCTACCA |
| **NEUROD1** | AGACACTCGTCTGTCCAGCTT |
| | GCCCCAGGGTTATGAGACTA |
| **GDNF** | CGCCGAAGACCGCTCCCTCG |
| | ATCCATGACATCATCGAACTGATC |
| **RET** | GTCCTCTTGCTCCACTTCAACG |
| | CCTGGCAGTTTTCCACACAGAC |
| **NTRK2** | ACAGTCAGCTCAAGCCAGACAC |
| | GTCCTGCTCAGGACAGAGGTTA |
| **VGF** | ACCCACAGCCTGGATAACAG |
| | ACTGGTTGCCATCAAACTCC |
| **GRB2** | GAAATGCTTAGCAAACAGCGGCA |
| | TCCATCTCGGAGCACCTTGAAG |
| **RASD2** | GACATCCTGGATACCTCTGGCA |
| | CGGTTATCCAGGCTGAACACCA |
| **CAT** | GTGCGG AGATTCAAC ACTGCCA |
| | CGGCAATGTTCTCACACAGACG |
| **GPX7** | CGACTTCAAGGCGGTCAACATC |
| | TCGGTAGTGCTGGTCTGTGAAG |
| **GRIA1** | GGATGCTCTTTCAGGACCTGGA |
| | GTAGTGGTAGCCGATGCCATTC |
| **GRIA2** | CCTGGATTCCAAAGGCTATGGC |
| | GTCTAAGACGCCTTGCTCACTG |
| **GRIA3** | GGTTCAGCAGTTCATACAGCC |
| | CTCCTCAGGTAGCGGAAAGCTT |
| **GRID1** | AGGAAGGCTACCTCCAGATGCT |
| | CAGTTGAGGCTCGCCATGCTAT |
| **GRID2** | TCTTACACGGCAAACCTCGCTG |
| | TACCGCAGAGTCTAGGACTGTG |
| **GRIK4** | ATGGACAGCCACCTCTATGCCT |
| | GTAGCGGTCATTGCCTTCCATC |
| **GRIN2B** | TTCCACTGGCTATGGCATTGCC |
| | GACAAATGCCAGTGAGCCAGAG |
| **SLC1A2** | TGCCAACAGAGGACATCAGCCT |
| | CAGCTCAGACTTGGAGAGGTGA |
| **BAX** | TCAGGATGCGTCCACCAAGAAG |
| | TGTGTCCACGGCGGCAATCATC |
| **SOD1** | CTCACTCTCAGGAGACCATTGC |
| | CCACAAGCCAAACGACTTCCAG |
| **SOD2** | CTGGACAAACCTCAGCCCTAAC |
| | AACCTGAGCCTTGGACACCAAC |
| **CYCS** | AAGGGAGGCAAGCACAAGACTG |

| | |
|---|---|
| | CTCCATCAGTGTATCCTCTCCC |
| **GRIK1** | GGATGTATGTGCTCTTAGCCTGC |
| | GTTTTCCACCACGTCTGAGTCAG |
| **GFRA1** | CATAGACTCCAGTAGCCTCAGTG |
| | GTCACATCGGAGCCATTGCCAA |
| **CYGB** | ACCGCTGCCTACAAGGAAGTGG |
| | AAGGAGGGTCTTCAGAACTCGG |
| **NQO1** | CCTGCCATTCTGAA GGCTGGT |
| | GTGGTGATGGAAAGCACTGCCT |
| **HOXB5** | GACTCCGCAAATATTCCCCTGG |
| | GGAACTCCTTTTCCAGCTCCAG |
| **PHOX2B** | CCTGAAGATCGACCTCACAGAG |
| | TTTTGCCCGAGGAGCCGTTCTT |
| **NKX2.1** | CAGGACACCATGAGGAACAGCG |
| | GCCATGTTCTTGCTCACGTCCC |
| **GAPDH** | GTCTCCTCTGACTTCAACAGCG |
| | ACCACCCTGTTGCTGTAGCCAA |
| **18S** | ACCCGTTGAACCCCATTCGTGA |
| | GCCTCACTAAACCATCCAATCGG |

| **Antibody** | **Vendor** | **Catalog #** | **Dilution** |
|---|---|---|---|
| mouse anti-Olig2 | Millipore | MABN50 | 1:1000 |
| rabbit anti-HB9 | DSHB | AB2145209 | 1:300 |
| rabbit anti-Islet1 | DSHB | AB528315 | 1:300 |
| Goat anti-ChAT | Millipore | AB144P | 1:200 |
| rat anti-VGlut1 | SYSY | 135311 | 1:300 |
| rabbit anti-GAD67 | Millipore | Mab5406 | 1:300 |
| rabbit anti-TUJ1 | Cell Signaling Technology | 5568 | 1:500 |
| mouse anti-TUJ1 | Cell Signaling Technology | 4466 | 1:500 |
| rabbit anti-Synapsin 1 | Cell Signaling Technology | 2312 | 1:200 |
| rabbit anti-pRET | R&D systems | AF5009 | 1:1000 |
| rabbit anti-RET | Cell Signaling Technology | 14556 | 1:1000 |
| rabbit anti-CAT | Cell Signaling Technology | 12980 | 1:1000 |
| rabbit anti-GPX7 | GeneTex | GTX105683 | 1:1000 |
| rabbit anti-VGF | Origene | TA306578 | 1:1000 |
| rabbit anti-pERK | Cell Signaling Technology | 9101 | 1:1000 |
| mouse anti-tERK | Cell Signaling Technology | 4696 | 1:1000 |
| rabbit anti-pAkt | Cell Signaling Technology | 4060 | 1:1000 |
| rabbit anti-tAkt | Cell Signaling Technology | 9272 | 1:1000 |
| rabbit anti-pSrc | Cell Signaling Technology | 6943 | 1:1000 |
| rabbit anti-Src | Cell Signaling Technology | 2109 | 1:1000 |
| goat anti-GFRA1 | R&D systems | AF714 | 1:1000 |
| rabbit anti-GRIA1 | Cell Signaling Technology | 8652 | 1:1000 |

| | | | |
|---|---|---|---|
| Mouse anti-OCT4 | Stem Cell Technology | 60093.1 | 1:200 |
| Mouse anti-O4 | R&D systems | MAB1326 | 1 µg/mL |
| mouse anti-Actin | Proteintech | 66009-1 | 1:2000 |
